# Fabrication of Chemofluidic Integrated Circuits by Multi-Material Printing

**DOI:** 10.3390/mi14030699

**Published:** 2023-03-22

**Authors:** Alexander Kutscher, Paula Kalenczuk, Mohammed Shahadha, Stefan Grünzner, Franziska Obst, Denise Gruner, Georgi Paschew, Anthony Beck, Steffen Howitz, Andreas Richter

**Affiliations:** 1 Institute of Semiconductors and Microsystems, Technische Universität Dresden, 01062 Dresden, Germany; 2 Institute of Clinical Chemistry and Laboratory Medicine, University Hospital Carl Gustav Carus, Fetscherstr. 74, 01307 Dresden, Germany; 3 GeSiM—Gesellschaft für Silizium-Mikrosysteme mbH, Bautzner Landstrasse 45, D-01454 Radeberg, Germany

**Keywords:** chemofluidics, microfluidics, hydrogel, PEG, closing and opening valve, printing

## Abstract

Photolithographic patterning of components and integrated circuits based on active polymers for microfluidics is challenging and not always efficient on a laboratory scale using the traditional mask-based fabrication procedures. Here, we present an alternative manufacturing process based on multi-material 3D printing that can be used to print various active polymers in microfluidic structures that act as microvalves on large-area substrates efficiently in terms of processing time and consumption of active materials with a single machine. Based on the examples of two chemofluidic valve types, hydrogel-based closing valves and PEG-based opening valves, the respective printing procedures, essential influencing variables and special features are discussed, and the components are characterized with regard to their properties and tolerances. The functionality of the concept is demonstrated by a specific chemofluidic chip which automates an analysis procedure typical of clinical chemistry and laboratory medicine. Multi-material 3D printing allows active-material devices to be produced on chip substrates with tolerances comparable to photolithography but is faster and very flexible for small quantities of up to about 50 chips.

## 1. Introduction

One of the central success factors of microelectronic circuit technology is the coupling of the number of active chip components and thus of circuit functionality due to improvements in manufacturing technologies [1]. Layer structuring processes play a key role as they are beneficial to manufacture all important functional structures including metallic conductor paths, dielectrics and also the electronic semiconductor components by stacking structured material layers. Modern microelectronic circuits with billions of transistors go through several hundred sequential layer structuring processes, with pattern transfer almost exclusively by photolithography. It is natural to apply the economy of scale of a circuit technology to the diverse applications of microfluidics [2,3]. However, although now mature and economically successful, lab-on-a-chip (LoC) achievements to date have hardly been based on circuit scaling. In the few commercial large-scale integrated (LSI) LoC, for example, from Fluidigm, photolithographic layer structuring processes play a major but not scaling role. The most important reason for this is probably the lack of a universal component such as the microfluidic transistor, on which an economy of scale can be based [4]. Logical microfluidics [5,6] addresses this issue with its two manifestations: pressure-controlled logical micropneumatics with membrane-based switching elements, and chemofluidics. The latter differs from the other LoC platforms in that it is not controlled electronically but directly by the process chemistry on the chip and is based on active-material devices, which are also to be integrated into the chip by layer-structuring processes [7,8,9]. Already in the first reports, LSI circuits with active-material components based on three different stimulus-sensitive polymers integrated by different technologies including mask printing, photolithography and pick-and-place were described [10,11]. Meanwhile, there is a wide variety of work on photolithographic patterning and integration of hydrogel components [12,13,14,15] both as in situ polymerization of gels within a microfluidic chip and as a process prior to the chip assembly [16,17,18,19,20]. However, photolithography for chemofluidic LoC is very challenging, especially at lab scale for small quantities of 5 to 50 chips. There are three main reasons for this. Firstly, standard photolithographic equipment such as steppers or mask aligners have to be converted to realize an inert gas atmosphere and to expose chamber systems for the prepolymer solution to be exposed, or specially designed exposure systems have to be used [20]. Secondly, extensive mask sets are needed [21,22] because individual masks are required for each active material layer but also for devices with different cross-linking or polymerization parameters. Thirdly, chemofluidic ICs are currently large-area chips with dimensions that may be as large as 10 cm in one dimension. It is therefore a challenge to ensure photolithographically that all active-material components have the same component tolerances distributed over the entire chip area; in addition, positional errors of the individual active-material structures in the valve chambers should be largely avoided [21,22]. Since the large chips also largely prevent parallel batch production, the laboratory production of a single chip can take several days.

An alternative patterning technique for active and soft polymers is 3D printing. In particular, 3D printing of hydrogels has been well studied due to its relevance to tissue engineering. It can be performed using stereolithography, digital light printing, and extrusion-based techniques [23,24,25,26,27]. Here, we report on a manufacturing process of chemofluidic circuits based on multi-material printing that is an alternative to photolithography, using the example of two different materials, meltable polyethylene glycols and photopolymerizable hydrogels.

## 2. Materials and Methods

### 2.1. Materials

The following materials were used for chip production: Poly(methyl methacrylate) (PMMA) foils with a thickness of 250 µm and 175 µm (Plexiglas 99524, Röhm GmbH, Darmstadt, Germany), hydrophilic polyester (PE) foil with a thickness of 170 µm (9960 Diagnostic Microfluidic Hydrophilic Film, 3M, Maplewood, MN, USA), adhesive transfer tape with a thickness of 25 µm (9969 Microfluidic Diagnostic Tape, 3M, USA), single-sided silicone adhesive tape with a thickness of 100 µm (9795R Microfluidic Diagnostic, 3M, USA), hydrophobic polytetrafluoroethylene (PTFE) membrane filters with a thickness of 100 µm and pore size of 1.2 µm (11803-25-N, Sartorius AG, Göttingen, Germany).

Red ink (Kombi, ONLINE Schreibgeräte GmbH, Neumarkt, Germany) and blue ink (Pelikan Group GmbH, Hannover, Germany) were used for chip testing. To prepare the ink solutions, the blue ink was diluted 24.5 times and the red ink was diluted 41 times.

Materials for hydrogel synthesis include *N*,*N*-dimethylacrylamide (DMAA, Sigma-Aldrich, St. Louis, MO, USA), *N*,*N*′-methylenebisacrylamide (BIS, Sigma-Aldrich, St. Louis, MO, USA) and photoinitiator lithium-phenyl-2,4,6-trimethylbenzoylphosphinate (LAP, Bio-Techne GmbH, Minneapolis, MN, USA). As chromatographic column material, basic aluminum oxide (Acros Organics, Geel, Belgium) was used. The material base for soluble opening components is polyethylene glycol with an average molecular weight of 35,000 g/mol (PEG 35,000, Merck, Darmstadt, Germany).

### 2.2. Equipment

#### 2.2.1. Multi-Material Printing Station

**Figure 1:**
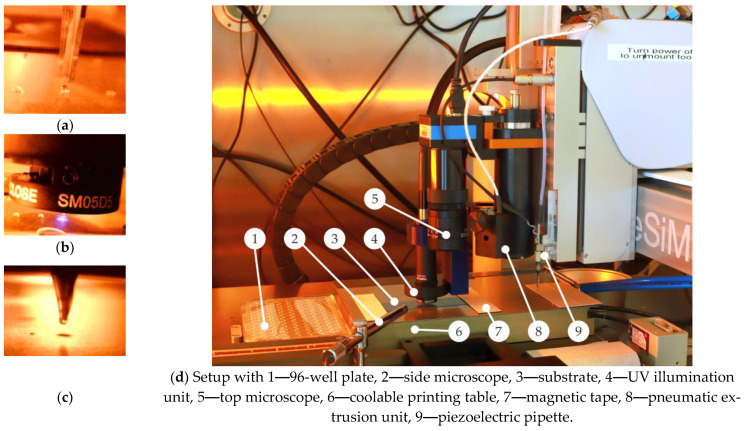
Printer setup under protective nitrogen atmosphere inside the glovebox; (**a**) process of inkjet printing of hydrogel prepolymer solution; (**b**) UV exposure of the prepolymer solution, (**c**) pneumatic extrusion of melted PEG for opening-valves and (**d**) an overview of all tools. The supporting video material (Videos S1 and S2) gives further insight.

The multi-material printing station is a specific system development together with GeSiM mbH. The printer unit is based on the 3D prototyping printer BioScaffolder 3.2 (GeSiM mbH, Radeberg, Germany), which can be equipped as a platform system with a variety of different material deposition tools (Figure 1). In the configuration used here, it is equipped with two different printing tools. The first printing tool is a piezoelectric pipette (Nano-Tip HV-J) for contactless spotting of low-viscosity inks, here hydrogel prepolymer solutions. The second printing tool intended for contact mode printing is a heatable cartridge with a metallic nozzle of an inner diameter of 100 µm. The dosing of the molten materials to be printed is performed pneumatically. During material dispensing, the print head can be moved three-dimensionally so that 1D, 2D and 3D printing can be performed along a path.

For UV photopolymerization of the prepolymer droplets into hydrogel structures, the piezo-pipet unit is equipped with a UV exposure unit. The implemented UV exposure system OmniCure S1500A with a 200 W Mercury Arc Lamp (Lumen Dynamics Group Inc., Mississauga, ON, Canada) is equipped with a 320–500 nm filter to reduce damage to the polymer material. In addition, to restrict the exposed area, an optical field diaphragm is built in using SM05D5 iris with a diameter between 0.6 mm and 5 mm. Spotted prepolymer solution can be sequentially exposed as a single dot or as a group of dots by time control via an electric shutter.

An observation camera with high magnification but small depth of field is also attached to the moving head to control the x-y positions. The small depth of field enables an optical z-value detection of the top side of the sample by observing the sample contrast and tracking the setting of the print plane definition. Additionally, a pen-type microscope is integrated at the print field for side-view verification of the process flow.

The sample holder not only has the purpose of fixing the sample in a defined position but also of keeping it at the right temperature. The temperature control of the material during deposition affects, on the one hand, the cooling rate for molten materials and, on the other hand, the evaporation rate and the material crosslinking/polymerization parameters in the case of reactive material printing. Therefore, the selected vacuum chuck is equipped with a liquid cooling. The temperature control of the liquid with a hysteresis of ±0.2 K is provided by an external thermostat ARCTIC A10 (Thermo Scientific, Waltham, MA, USA). An additional magnetic tape allows the straight clamping of the sample onto the holder.

The multi-material printer is integrated into a glovebox (GS GLOVEBOX Systemtechnik GmbH, Malsch, Germany), whose glass panels are coated with a photolithography-compatible yellow filter film. Reactive polymer printing with a reproducibility that is appropriate for the high demands of circuit technology requires the conditions of an oxygen-free inert gas atmosphere [22].

During the whole flight time, the small droplets of reactive monomers are exposed to the surrounding gas atmosphere. Here we run the glovebox with nitrogen as protective gas, reducing the oxygen concentration below 100 ppm, and a relative humidity level of around 62%. For regular equipment maintenance, the front windows of the chosen glovebox can be pneumatically unlocked and opened.

#### 2.2.2. Excimer ExciJet172 55-130 Lab System

The excimer ExciJet172 55-130 UV lamp is a standard equipment of Ushio Germany GmbH. The pretreatment of the substrates was performed at 172 nm and 10 mW/cm^2^.

#### 2.2.3. Compact Laser Micromachining System RDX500

The micromachining station is a specific system developed with Pulsar Photonics GmbH based on the standard RDX500 system (Pulsar Photonics GmbH, Herzogenrath, Germany), additionally configured with a femtosecond UV source (Pharos PH1-10, 343 nm, 280 fs-10 ps, 2.5 W, Light Conversion, Vilnius, Lithuania) and a circular polarization within the beam forming and a 100 mm telecentric objective.

#### 2.2.4. Microfluidic Test Station

Electric pneumatic pressure controller (series ITV0000, SMC Corporation, Tokyo, Japan), miniaturized solenoid pressure valves (valves manifold MH1, 16 valves, Festo Corporation, Esslingen, Germany), magnetic flow valves (Buerkert GmbH, München, Germany), electric control unit (WAGO-I/O-System Fieldbus 750–881), flow sensor (SLI-1000, Sensirion Holding AG, Stäfa, Switzerland). Microfluidic pipe (ND-100-80, inner diameter 0.25 mm, Saint Gobain S.A., Paris, France), 100 mL glass bottle (Labsolute, Th. Geyer GmbH, Renningen, Germany), Screw cap distributor for bottles (F746, Bola, Bohlender GmbH, Grünsfeld).

### 2.3. Application Chip

#### 2.3.1. Preparation of Prepolymer Solution for Hydrogel Printing

The active hydrogel component was synthesized from commercially available monomer DMAA, which was beforehand filtered through a basic aluminum oxide chromatographic column to remove the stabilizer. The prepolymer solution was prepared by mixing the monomer DMAA (concentration 1.45 mmol/L), the crosslinker BIS (0.09 mmol/L) and the photoinitiator LAP (0.002 mmol/L) in deionized water, then stirred and purged with argon for 10 min. The solution was stored in a sealed flask and protected from UV light. Finally, for printing purposes, 50 µL of the prepolymer solution was filled into one well of a 96-well plate and aspirated into the piezo-pipette for subsequent deposition.

#### 2.3.2. Chip Manufacturing

**Figure 2:**
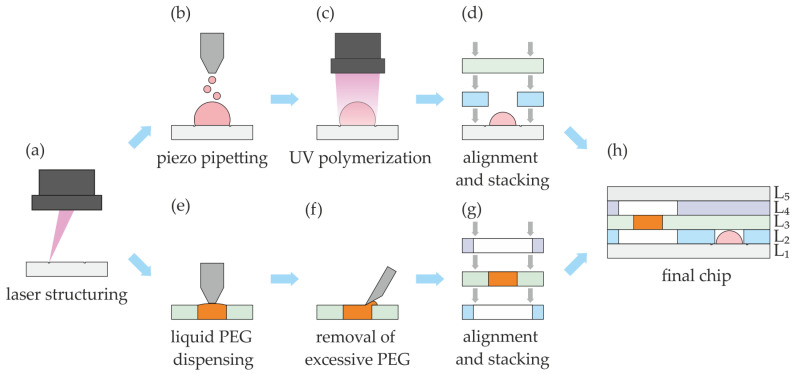
General process flow of fabrication of multilayer chemofluidic ICs with multi-material printing. The different valve types are manufactured in separate layers (upper process flow (**b**–**d**) for hydrogel-based closing valves; lower process flow (**e**–**g**) for soluble PEG opening valves; (**a**) laser structuring of the polymer layers, including engraving; (**b**) ink-jet printing of prepolymer solution; (**c**) UV exposure of prepolymer solution; (**d**) alignment, stacking and bonding of the closing valve sub-stack; (**e**) dispensing of molten PEG; (**f**) removal of excessive PEG; (**g**) alignment, stacking and bonding of the opening valve sub-stack; and (**h**) combination of the sub-stacks to the complete chip with L_1_ + L_3_ PMMA foil, L_2_ + L_4_ PE foil with adhesive 9960 on both sides, L_5_ PMMA foil with 9795R on top side.

The general process flow of chip manufacturing is shown in Figure 2.

The chip has a multilayer architecture consisting of several laser-structured polymer foils bonded together using adhesive tape (PMMA foils for the valve substrates, PE foils laminated with adhesive tape for intermediate layers, PMMA foil laminated with silicon-based adhesive for the top layer). Laser structuring of the foils (Figure 2a) with the RDX-500 takes place at a speed of 500 mm/s and a peak fluence of 11.03 J/cm^2^. For the hydrogel valves, phase guides were engraved with a depth of about 100 µm (marking speed of 100 mm/s with a fluence of 5.09 J/cm^2^) on the PMMA foils L_1_. After valve layers L_1_ and L_2_ have been manufactured, they are aligned with guide pins in a special device and bonded together by pressing (Figure 2e) to form the final chip. The complete chip is finally equipped with the fluidic chip-to-world connections. Due to the difference between the two active materials, the valve layers are fabricated using different procedures, which are described below in separate sections.

#### 2.3.3. Fabrication of Hydrogel-Based Closing Valves

**Figure 3:**
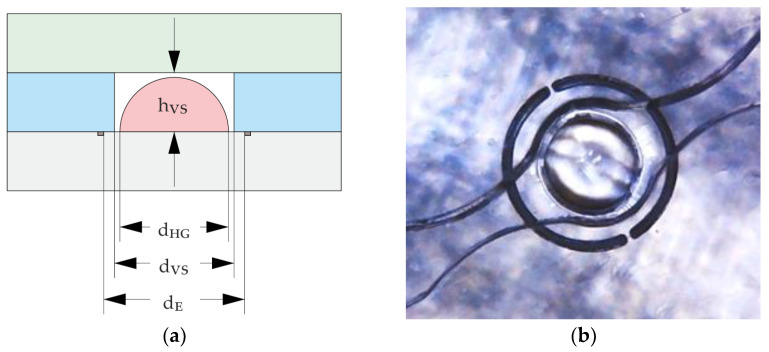
(**a**) Schematic cross-section of closing valve layer stack; pink—hydrogel element, blue—fluidic layer; the width of the hydrogel, d_HG_; the diameter of the valve seat, d_VS_; the diameter of the engraving, d_E_; thickness of the foil of fluidic layer, h_VS_. (**b**) Top view of dry hydrogel positioned in 475 µm diameter (d_VS_) valve seat and 600 µm diameter (d_E_) engraving.

By using the piezo pipette, the prepolymer solution is printed onto the midpoint of the engraved phase guides of the PMMA foil (Figure 2b). The engraved phase guides define the footprint of the prepolymer solution and the subsequent hydrogel structures. The engraved phase guide is not continuous to avoid fluid leakage flow. They also compensate for the angular error in the ideal flight path of the droplets as they leave the pipette nozzle due to contamination or bubbles inside the pipette. In addition, the foils are less hydrophilic to achieve better volume per area deposition and prevent wetting of the foil at undesired areas. To prevent spotting outside the engraved area, the distance between pipette and substrate was being changed stepwise during droplet deposition, starting with 0.4 mm for the first 50 droplets, then 0.6 mm for the next 150 droplets and additional +0.2 mm for each next 200 droplets, ensuring that the pipette is close to the dispensed volume at all times. For the valve configuration shown in Figure 3, the volume of the printed prepolymer solution is 450 ± 10 pl per droplet and 400 droplets in total. The following parameters of the piezo-pipette were used: voltage between 40 V and 60 V, pulse width of 90 µs and frequency of 100 Hz.

After depositing the desired prepolymer solution volume, it was exposed to UV light (Figure 2c). The printing table was cooled down to near the dew point of about 13 °C to prevent evaporation of water and shrinkage of the deposited shape of the liquid prepolymer solution. After the gels have dried in the horizontal substrate position, the substack can be produced (Figure 2d).

#### 2.3.4. Fabrication of PEG-Based Opening Valves

**Figure 4:**
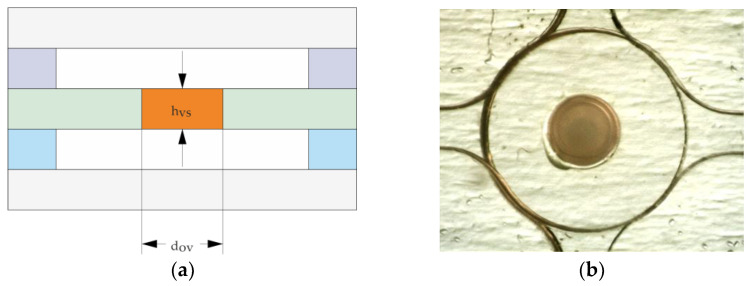
(**a**) Schematic cross-section of opening valve layer stack; orange—PEG element, blue and violet—fluidic layers.; thickness of the foil with the hole, h_VS,_ and diameter of the hole, d_OV_. (**b**) Top view of cured PEG element positioned in 500 µm hole (d_OV_) and placed between microfluidic layers.

PEG elements Figure 4, lower process flow Figure 2f–h) acting as opening valves were printed with the heatable pneumatic cartridge dispenser into holes of different diameters (125 µm, 250 µm and 500 µm) structured in PMMA foils (175 µm or 250 µm thick). The foils were put on a frame of PDMS with a thickness of around 5 mm. This ensured that the metallic nozzle could freely move to achieve complete filling of holes due to surface tension. For melting the PEG 35,000, the cartridge was heated to 90 °C, and the additional nozzle heater was heated to 92 °C. The pressure for the pneumatic dispenser was set to 80 kPa and activated for 30 s, 10 s and 6 s for 500 µm, 250 µm and 125 µm holes, respectively. For the 500 µm hole, the nozzle could move inside the hole to wet the whole circumference. For the 250 µm and 125 µm holes, the outer diameter of the nozzle was too large so that the nozzle could only touch the surface. A sufficient amount of PEG was dispensed so that the complete filling of the laser-drilled holes was ensured. Afterwards, the excess PEG was manually removed with a sharp blade and subsequently the protective foil for further stacking of layers.

#### 2.3.5. Test Station to Characterize the Opening Valves, Closing Valves and Application Chip

The test station used to characterize opening valves and closing valves and the application chip utilizing air pressure are described in [28]. The same measurement setup that Frank et al. 2016 built was used in this work, except the pressure sensor was not integrated in the station. All components of the test station are listed in Section 2.2.4. The opening and closing valves were characterized at a pressure of 80 mbar and a flow rate of 70 µL/min, while the application chip was operated at a pressure of 50 mbar and a flow rate of 40–45 µL/min to prevent air bubbles inside the chip.

#### 2.3.6. Design of the Application Chip and IC Program

**Figure 5:**
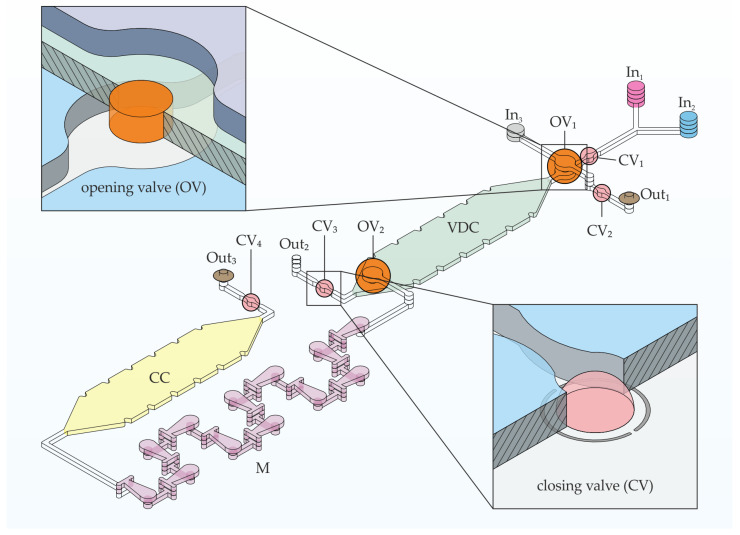
An isometric projection demonstrating the building elements and their arrangement in the application chip for volume definition, incubation and mixing of two liquids. The chip was constructed of six polymeric layers. Two insets showing isometric cross-section cutaways of closing and opening valve. M—3D micromixer, CV—closing valve, OV—opening valve, In—inlet, Out—outlet, VDC—volume definition chamber, CC—collecting chamber. The brown discs at Out_1_ and Out_3_ represent the integrated PTFE membranes.

The chemofluidic chip automates an analytical procedure that is essential in clinical chemistry and laboratory medicine: a sample is analyzed for a specific clinically relevant parameter. For that, any fluidic protocol must first accurately dose and then reliably mix the volumes of sample and detection reagent to record reaction kinetics or perform an end-point analysis. To automate it, conventional microelectromechanical lab-on-a-chip platforms require not only a special microfluidic chip but also transducers that control the chip components, a computer and control software [29,30]. Chemofluidic ICs are complete systems that do not require any external control and electronics; here, we only use external constant pressure sources providing 50 mbar to introduce the liquids into the chemofluidic IC. The program of the chemofluidic IC (Figure 5) automates the following workflow:

(1) Volume definition of two liquids in a volume definition chamber (VDC) of a total volume of 11 µL. The constant pressure source pumps liquids 1 and 2 simultaneously from inlets In_1_ and In_2_ at a flow rate of 40–45 µL/min into the VDC chamber, which fills with 5.5 µL of each liquid. Air and excess liquid escape via Out_1_ and Out_2_. When wetted with liquid, the active components are activated. The identical valves CV_1_ to CV_3_ (valve chamber diameter 475 µm, 450 pl per droplet and in total 400 droplets printed prepolymer solution) close after 1.5 min and lock VDC. At the same time, OV_1_ (hole diameter 500 µm, PMMA foil thickness 250 µm) opens and connects the inlet In_3_ to VDC.

(2) Incubation of the two fluids in VDC for a certain period of time. The opening valve OV_2_ (hole diameter 500 um, PMMA foil thickness 250 µm) opens after 5 min.

(3) Transport of liquids 1 and 2 by liquid 3 (transport liquid). The constant pressure moves liquid 3 into VDC and drives liquids 1 and 2 through a passive 3D mixer (M) [31] into the collection chamber (CC) of a volume of 11 µL; 1.5 min after activation, CV_4_ closes, which also prevents evaporation of the liquid when heated for analytical purposes.

**Figure 6:**
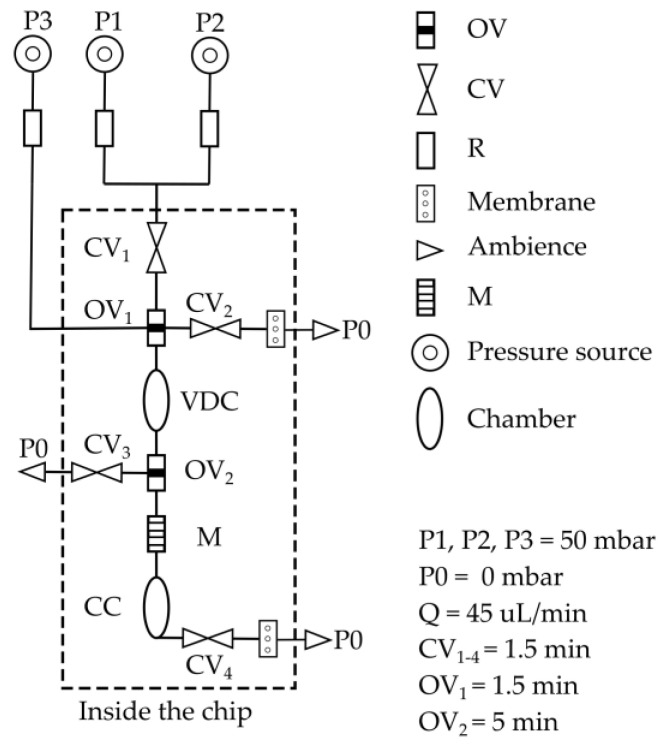
Circuit schematic of the application chip showing the building elements and their arrangements. M—3D micromixer, CV—closing valve, OV—opening valve, VDC—volume definition chamber, CC—collecting chamber, P—pressure, R—resister, Q—flow rate. The opening and closing times of all valves are also indicated. The resister (R) was a 60 cm tubing of a diameter of 0.25 µm provided so high resistance that the resistance caused by the microfluidic building elements was negligible and the flow rate in the chip remained constant.

In addition to integrated valves based on active materials, air exhaustion valves based on a hydrophobic air permeable PTFE membrane were also integrated between layer 4 and 5 at Out_1_ and Out_3_ to enable an immediate closure of the flow. Actual flow stop was required at Out_1_ and Out_3_. A PTFE membrane was not integrated at Out_2_ to prevent the immediate closure of VDC, allowing the formation of two laminar flows of liquids 1 and 2. A circuit schematic of the application chip is shown in Figure 6.

## 3. Results

The investigations focus on the influence of the manufacturing parameters on the properties of the printed components and their usability as circuit components. Finally, the application chip is investigated with regard to its basic functionality.

### 3.1. Characterization of the Hydrogel-Based Closing Valves

As shown in Section 2.3.6, chemofluidic valves have two functions, firstly the fluidic function as a switching element and secondly a time definition function, because the chemofluidic components also determine the time sequence of the IC program in their interconnection [10]. The component properties and tolerances are determined by a whole series of design parameters including mechanical closing condition, shape, and position of the hydrogel structures, the chemical composition and synthesis parameters [32,33,34].

#### 3.1.1. Design Parameters

**Figure 7:**
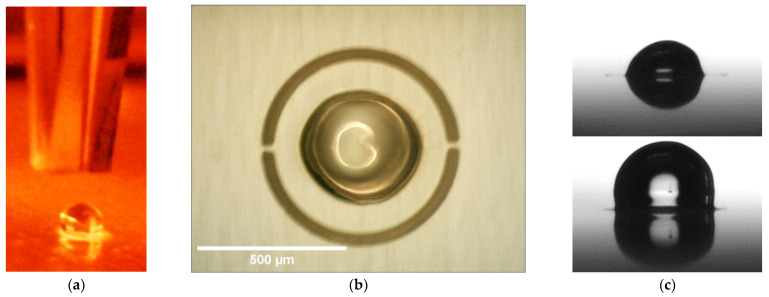
(**a**) Side view during printing process, the upper part depicts the tip of the piezoelectric pipette and underneath it the deposited hydrogel prepolymer solution (400 droplets with a volume around 450 pl per drop) inside the circular engraving on a PMMA substrate. (**b**) Top view of a printed hydrogel (60 s UV exposure time, 400 droplets, ca. 450 pl per drop) after drying, positioned in 600 µm diameter (d_E_) engraving. (**c**) Side views of a dry hydrogel (**top**) and of a swollen hydrogel (**bottom**), with the same synthesis parameters as in (**b**).

The most important design parameters are the shape of the hydrogel droplets, the hydrogel volume and the position of the hydrogel structure in the valve chamber. During the piezoelectric pipette printing (Figure 7a), a droplet consisting of a total of 400 individual droplets forms within the predefined engraved area, completely filling the area inside the lasered engraving. It has a pronounced spherical shape due to the narrow limitation and the hydrophobic substrate. Through optimized stepwise printing, a deposition rate of 100% (15 samples examined) was achieved for up to 600 droplets per hydrogel. A further increase in the number of droplets and thus the total droplet volume leads to printing losses (Table 1), as not all individual droplets contribute to the formation of the large droplet. As shown in Figure 7b the gels contracted into the center of the engraving as they gradually dried.

The hydrogel shrinkage resulted in circular-shaped hydrogels and showed a symmetrical behavior. Dry hydrogels were located in the middle of the engraved area and revealed only a small off-center deviation of 5 µm ± 2 µm. Such low misplacement deviation leads to the conclusion that these active elements can be printed with high precision and allows for avoiding positional errors in the entire chip.

As shown in Figure 7c, during drying and subsequent swelling, the hydrogel retains its significant expansion capacity, which enables a subsequent valve functioning. Moreover, the topography of the closing elements was investigated with a confocal scanning microscope µ-Surf (NanoFocus AG, Oberhausen, Germany). The obtained line profiles of five hydrogels (see Figure S1), going through their center points on an engraved foil, revealed that the height of the dried hydrogels equals about 174 ± 3 µm and the width equals about 192 ± 4 µm measured by light microscope (60 s UV exposure time, 400 droplets, ca. 450 pl per drop), so that here, too, there is a high degree of precision.

**Figure 8:**
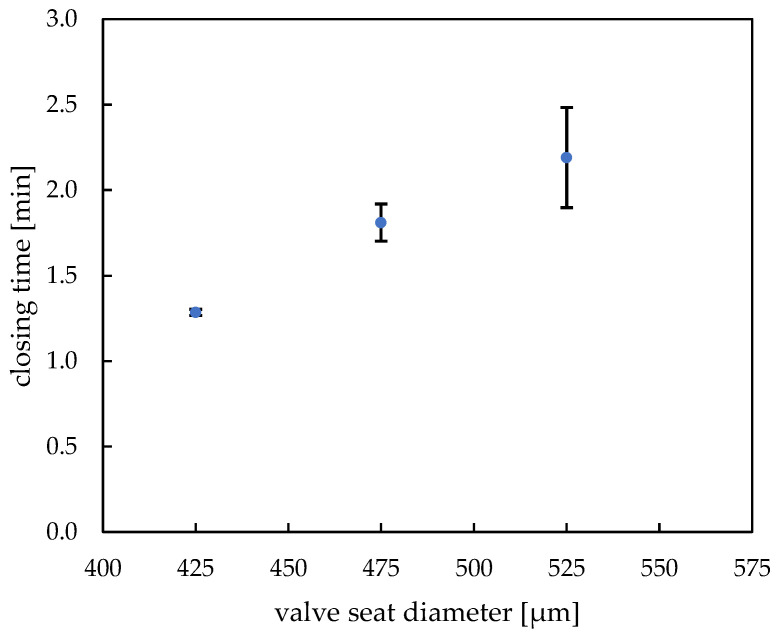
Dependence of hydrogel valve closing time on valve seat diameter (d_VS_) (60 s UV exposure time, 400 droplets, ca. 450 pl per drop).

The closing time of a closing valve is determined by the ratio of the dry volume of the hydrogel actuator to the valve chamber volume. If the dry hydrogel actuator is very small, it must swell for a long time to achieve the closing condition of hydrogel actuator volume equal to valve chamber volume. In Figure 8, the dependence of closing time on the valve seat size with constant engraving diameter (600 µm) in which closing elements have been printed on is shown (see Figure S2 for exemplary closing curve diagram). As expected, the closing time increases with increasing diameter of the valve seat; the actuators need to expand for longer time to fill up the entire valve volume and close it. Variation of only this one operating parameter can lead to different valve operation and, if needed, enables designing precise microfluidic protocols. The closing time for our standard hydrogel and valve seat diameters 425 µm to 525 µm lies within the range of 1 min 15 s to 2 min 10 s, which meets the needs of our chosen application chip. In this chip, the 475 µm valve seat with closing time of about 1 min 50 s has been implemented. In case of bigger valve seats (575 µm), the exact actuator position in the engraving proved to be a main factor during valve operation—since the hydrogels are the starting point for the layer alignment, even a slight deviation from their central position resulted in an adverse layer rearrangement. Since both the engraving and the valve seat are of similar dimension, their displacement resulted in unrestricted fluid flow, regardless of the successful actuator functioning (see Video S3). It is to be noted that the closing time deviation increases noticeably with higher valve seat diameter. As the closing time depends strongly on the position of the active hydrogel element in the valve and the “active element:valve seat diameter” ratio, it is to expected that at smaller ratios the positional errors are higher and lead to more variable closing times. To avoid that, it would be well advised to keep the mentioned ratio closer to 1 and regulate the opening times by changing the chemical and structural parameters of the hydrogel itself.

Since in our application the closing function is very important, the valve performance was further investigated in terms of its pressure resistance. The measurement was carried out in 100 mbar intervals in two differently sized valves (425 µm and 500 µm diameter valves) with pre-swollen hydrogels, starting from a pressure of 180 mbar. The hydrogels proved to be highly pressure resistant in case of both valve seat sizes, enduring the pressure up to 980 mbar without leakage (evaluated by a detected constant fluid flow rate of maximum 1.7 µL/min throughout the whole experiment). The pressure resistance depends on the degree of filling of the valve chamber with dry hydrogel. The higher the degree of filling, the higher the pressure resistance of the valves [32].

#### 3.1.2. Synthesis Parameters

The method of hydrogel actuator printing uses the surface tension of the liquid to obtain a continuous and smooth shape and maximize filling. During the UV exposure, the chain growing and the crosslinking reaction takes place, building up the solid hydrogel materials for the valve.

According to the processes of building up the gel material and binding the material to the substrate, the dry state volume differs. Further, according to the binding forces of the hydrogel body to the substrate, the gel can more or less freely reduce the radius during the shrinking process.

**Figure 9:**
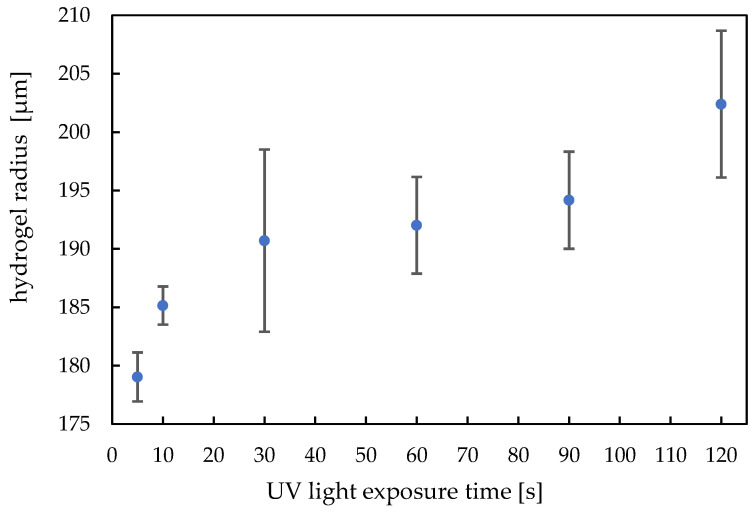
Dependence of the radius of hydrogels in dry state on the UV light exposure time (400 droplets, ca. 450 pl per drop).

For crosslinked polymers such as hydrogels, by varying the dose, the chain length and the degree of crosslinking can be adjusted. This affects the Young’s modulus, dry volume and swelling capacity, as well as binding to the substrate. Figure 9 shows the dependence of the hydrogel radius in the dry state on the UV light exposure time during the polymerization step.

The dry gel diameter follows a trend: with a higher UV-dose, the dry state radius increases. The buildup reaction can be clearly observed for an exposure time below 30 s. Above this value, we can still observe a trend of increasing hydrogel radius with higher exposure dose. Up to 120 s, we do not observe polymer degradation which means we still have a clear gel body and the dry state radius has a highest value. The standard deviation for each UV exposure time consists of 9 values.

**Figure 10:**
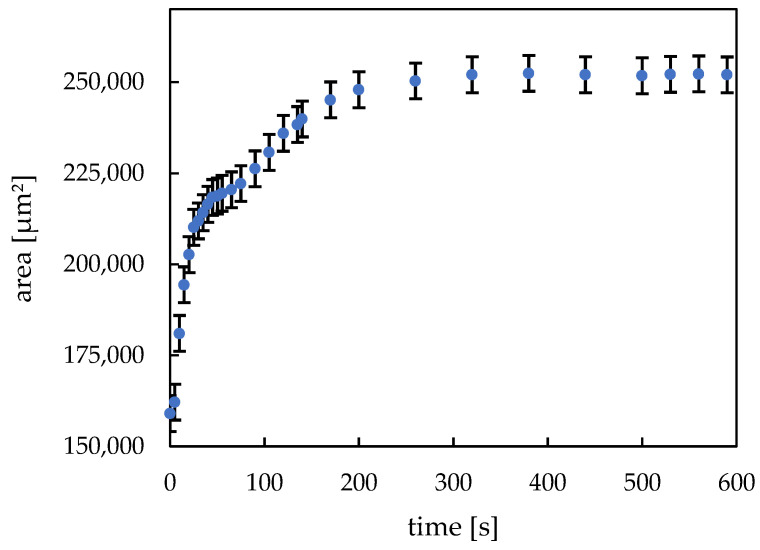
Free swelling of hydrogels (60 s UV exposure time, 400 droplets, ca. 450 pl per drop). The average hydrogel area results from three swelling kinetic measurements.

Figure 10 depicts the swelling kinetics of the printed hydrogel dots. The dried hydrogel dots immediately started swelling after they were immersed in deionized water. After approx. 25 s, the exponential swelling process stagnates for a short time and then continues at a slower rate until it reaches a state of equilibrium after approx. 260 s. This swelling behavior can be attributed to a release of unpolymerized monomer residues and a reorientation of the polymer chains formed since the printed hydrogel dots were not equilibrated before the experiment. Another possible explanation could be a so-called “skin effect” [33,35,36,37]. At first, only the outer areas of the hydrogel swell, since the fluid diffusion is uninterrupted there. Then, the swelling agent must penetrate the swollen “skin” barrier before it reaches the inner core of the hydrogel. As a result of that, the stagnation in the swelling curve can be unmistakably observed. From the recorded video (see Video S4), it is clear that at about 25 s, the inner structure of the hydrogel changes, and from this point on, the gel swells slower in the lateral dimension. All in all, the free swelling curve shows high uniformity and the error bars represent the standard deviation based on 3 different measurements and they could be assessed as relatively small. The swelling rate and its repeatability are essential properties of a hydrogel since its functionality as valve element must be guaranteed. The good reproducibility of the valve function was proven by the presented investigations.

### 3.2. Characterization of the PEG-Based Opening Valves

In this chapter, we focus on chemofluidic valves based on soluble polymer, polyethylene glycol (PEG). In contrast to closing valves, they do not absorb water but go through a dissolution process, which is an irreversible step [38]. Contrary to hydrogels, they do not show continuous operation due to their functioning characteristics (abrupt opening). The fundamental factors influencing the valve behavior are among other the material type and its amount, the process medium used and its flow rate during valve operation. In this work, PEG with an average molecular weight of 35,000 g/mol has been used since it provided an optimal opening time for our application chip.

**Figure 11:**
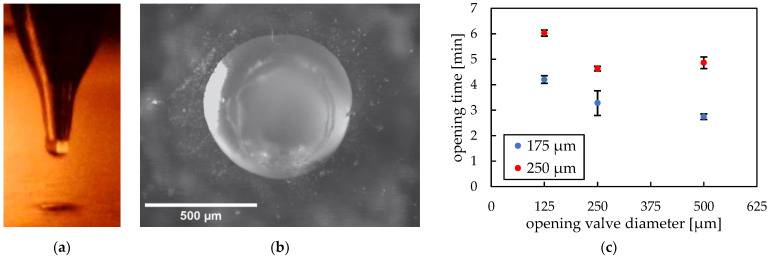
(**a**) Side view during pneumatic cartridge dispensing printing process, the upper part is the tip of the metallic nozzle of the pneumatic extruder and below is a 500 µm hole in a PMMA foil. (**b**) Top view of PEG printed and solidified in a hole. (**c**) Dependence of opening time on diameter of holes (d_OV_) and thickness (h_VS_) of PMMA foil.

Figure 11a shows a side view during the pneumatic cartridge dispensing process. The upper part is the tip of the metallic nozzle of the pneumatic extruder and below is a 500 µm hole in a PMMA substrate. A top view of PEG printed and solidified in a hole is depicted in Figure 11b.

Confocal scanning microscope line profiles of PEG opening valves after removing excess material and reflowing process showed a complete filling of the holes with a convex surface profile. Therefore, taking into consideration the resulting small height deviation, we could prove that the printing procedure is an exact and reliable method to produce such valves.

We investigated two important design parameters: diameter and thickness of the valve. Figure 11c shows the dependence of the opening time due to dissolving of water-soluble PEG positioned in differently sized holes (125 µm, 250 µm and 500 µm) in PMMA substrates either 175 µm or 250 µm thick. The ratios of opening hole diameter to foil thickness were either 0.7, 1.4 and 2.9 or 0.5, 1 and 2 for 175 µm and 250 µm thickness, respectively. The thinner PMMA substrate shows shorter opening time than the thicker substrate. This can be explained by the shorter dissolving time due to less material in the valve. The thinner PMMA substrate shows clear hole diameter dependence—the smaller the contact surface, the longer it takes for the valve to open. In contrast, the thicker PMMA substrate reveals a slight anomaly—the tests with 500 µm hole diameters resulted in longer opening times than the smaller sized holes. Considering that the relative material amount is significant, the PEG dissolving could have been impeded by the slow diffusion in the stagnant water, leading to longer opening times. To further investigate opening operation of the valve, PEG chain lengths could be varied or other soluble polymers such as poly(vinyl alcohol) (PVA) could be tested. The dissolution process is based on disturbed interaction between polymeric chains, which is why their length plays a significant role during the whole process [10,32]. This examination could possibly be carried out in our future ventures.

### 3.3. Application Chip

**Figure 12:**
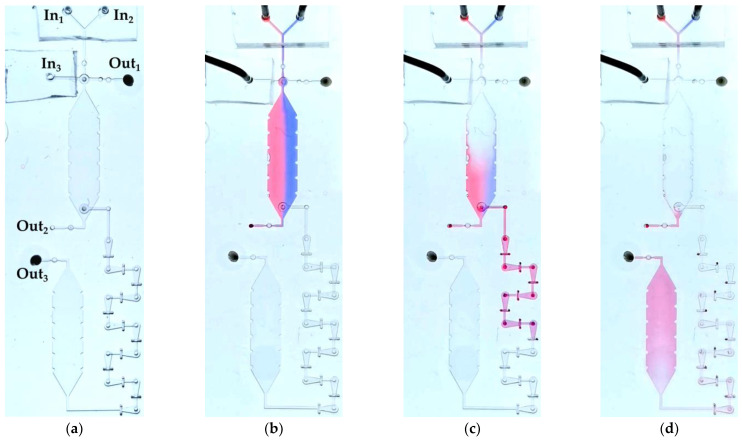
The chemofluidic application chip executes the circuit program. (**a**) An unfilled chip ready for operation showing the order of building elements. (**b**) Liquid 1 (red ink-stained water) and liquid 2 (blue ink-stained water) are simultaneously pumped into VDC at a constant pressure of 50 mbar and a flow rate of 40–45 µL/min. (**c**) Transportation of the two liquids by liquid 3 (unstained water) through M into CC (**d**).

The application chip depicted in Figure 5 and Figure 6 was tested with water as a process medium. It was the question of whether the IC-program to incubate and to mix defined volumes of two liquids was properly performed as planned in Section 2.3.6. Liquid 1 (red ink-stained water) and liquid 2 (blue ink-stained water) were pumped into VDC and their volumes were determined (Figure 12b). To ensure a homogenous filling of VDC and CC as well as to avoid bubble formation while filling, layer 1 and layer 3, which seal the chambers from top and bottom, were hydrophilized using excimer UV lamp. The unequal volumes of liquid 1 and liquid 2 can be explained by the slight difference in the viscosities between the two liquids. Red ink-stained water had a slightly lower viscosity than the blue ink-stained water. This led to pumping more red liquid into VDC than blue liquid. In about 1.5 min, OV1 opened and liquid 1 and liquid 2 did not move out through Out_2_ confirming the complete closure of CV_1_ and CV_3_. After an incubation time of about 5 min, OV_2_ opened and both liquids were driven by liquid 3 (unstained water) into M for mixing and finally into CC (Figure 12c,d). The opening valve (OV_2_) opened after 5 min and not after 1.5 min like OV_1_ because OV_2_ was wetted only from one side. This slowed down the total dissolving time of PEG and eventually led to a slower opening. In conclusion, the chemofluidic chip has successfully performed the fluidic IC-program as intended (Video S5).

## 4. Discussion

Multi-material inkjet 3D printing is proving to be a potent method for fabricating chemofluidic circuits, offering advantages over a photolithographic workflow, at least in laboratory production. Due to its concept, 3D printing has no problems ensuring consistent device tolerances even over large chip areas. We were also able to demonstrate that printing even very different materials (low-viscosity prepolymer solutions and meltable polymers) can be performed efficiently with just one machine. The production time of a chip in the lab is much shorter, the circuits can be realized in one day, which is a significant reduction compared to our photolithographic workflow, which takes several days per chip. In addition, material consumption is low and unnecessary contamination is largely prevented. Hydrogel-based closing valves can be printed precisely with suitable tolerances with regard to the component function. Particularly mentionable is a self-centering effect, which minimizes the positional errors of the hydrogel structures. Meltable opening valves can also be realized accurately and easily integrated into holes. In addition to chemofluidics, the entire field of hydrogel-based microtechnology can also benefit from high-performance multi-material printing, as it can be used to easily and precisely manufacture all components, including pumps [39,40], storage devices [41], filters [42,43], thermo- and chemostats [44,45,46], check valves [32,47,48] as well as microlenses [49] for optical systems and taxels [50] for tactile displays and microactuators [51,52], e.g., for soft robotics [53].

## Figures and Tables

**Table 1:** Yield of good hydrogels depending on the number of droplets (450 pl per drop) for 600 µm engraving (number of good hydrogels divided by 15 (total sample size for each number of droplets)). A yield of 100% means that all printed hydrogels can be used as closing valves for further processing.

Number of droplets	400	460	500	600	700	800	900	1000
Yield in %	100	100	100	100	73	93	47	33

## Data Availability

Not applicable.

## Supplementary Materials

The following supporting information can be downloaded at: https://www.mdpi.com/article/10.3390/mi14030699/s1, Figure S1: Line profiles of five hydrogels obtained with a confocal scanning microscope µ-Surf; Figure S2: Closing curve diagram for three hydrogels (400 drops, 60 s UV exposure time) in ventil seats of 425 µm diameter; Video S1: Printing with PEG into a hole with diameter 500 µm located in 175 µm thick PMMA foil; Video S2: Printing with prepolymer solution (450 drops with 550 pl each) on PMMA substrate with 600 µm diameter engraving; Video S3: Unrestricted fluid flow through engraving in spite of successful hydrogel actuator operation (575 µm diameter valve seat), caused by layer misplacement; Video S4: Free swelling of hydrogel (400 drops, 60 s UV exposure time) in water; Video S5: Recorded video of the fluidic IC-program of the application chip.

## References

[1] Hamilton S. (1999). Taking Moore’s Law into the next Century. Computer.

[2] Thorsen T., Maerkl S.J., Quake S.R. (2002). Microfluidic Large-Scale Integration. Science (1979).

[3] Melin J., Quake S.R. (2007). Microfluidic Large-Scale Integration: The Evolution of Design Rules for Biological Automation. Annu. Rev. Biophys. Biomol. Struct..

[4] Au A.K., Lai H., Utela B.R., Folch A. (2011). Microvalves and Micropumps for BioMEMS. Micromachines.

[5] Rhee M., Burns M.A. (2009). Microfluidic Pneumatic Logic Circuits and Digital Pneumatic Microprocessors for Integrated Microfluidic Systems. Lab Chip.

[6] Zhang Q., Zhang M., Djeghlaf L., Bataille J., Gamby J., Haghiri-Gosnet A.M., Pallandre A. (2017). Logic Digital Fluidic in Miniaturized Functional Devices: Perspective to the next Generation of Microfluidic Lab-on-Chips. Electrophoresis.

[7] Frank P., Schreiter J., Haefner S., Paschew G., Voigt A., Richter A. (2016). Integrated Microfluidic Membrane Transistor Utilizing Chemical Information for On-Chip Flow Control. PLoS ONE.

[8] Frank P., Gräfe D., Probst C., Haefner S., Elstner M., Appelhans D., Kohlheyer D., Voit B., Richter A., Frank P. (2017). Autonomous Integrated Microfluidic Circuits for Chip-Level Flow Control Utilizing Chemofluidic Transistors. Adv. Funct. Mater..

[9] Beck A., Mehner P.J., Voigt A., Obst F., Marschner U., Richter A. (2022). Logic Circuits Based on Chemical Volume Phase Transition Transistors for Planar Microfluidics and Lab-on-a-Chip Automation. Adv. Mater. Technol..

[10] Greiner R., Allerdissen M., Voigt A., Richter A. (2012). Fluidic Microchemomechanical Integrated Circuits Processing Chemical Information. Lab Chip.

[11] Allerdissen M., Greiner R., Richter A. (2013). Microfluidic Microchemomechanical Systems. Adv. Sci. Technol..

[12] Hoffmann J., Plötner M., Kuckling D., Fischer W.J. (1999). Photopatterning of Thermally Sensitive Hydrogels Useful for Microactuators. Sens. Actuators A Phys..

[13] Beebe D.J., Moore J.S., Bauer J.M., Yu Q., Liu R.H., Devadoss C., Jo B.H. (2000). Functional Hydrogel Structures for Autonomous Flow Control inside Microfluidic Channels. Nature.

[14] Obst F., Beck A., Bishayee C., Mehner P.J., Richter A., Voit B., Appelhans D. (2020). Hydrogel Microvalves as Control Elements for Parallelized Enzymatic Cascade Reactions in Microfluidics. Micromachines.

[15] Obst F., Simon D., Mehner P.J.J., Neubauer J.W.W., Beck A., Stroyuk O., Richter A., Voit B., Appelhans D. (2019). One-Step Photostructuring of Multiple Hydrogel Arrays for Compartmentalized Enzyme Reactions in Microfluidic Devices. React. Chem. Eng..

[16] Yu C., Mutlu S., Selvaganapathy P., Mastrangelo C.H., Svec F., Fréchet J.M.J.J. (2003). Flow Control Valves for Analytical Microfluidic Chips without Mechanical Parts Based on Thermally Responsive Monolithic Polymers. Anal. Chem..

[17] Simon D., Obst F., Haefner S., Heroldt T., Peiter M., Simon F., Richter A., Voit B., Appelhans D. (2019). Hydrogel/Enzyme Dots as Adaptable Tool for Non-Compartmentalized Multi-Enzymatic Reactions in Microfluidic Devices. React. Chem. Eng..

[18] Heo J., Crooks R.M. (2005). Microfluidic Biosensor Based on an Array of Hydrogel-Entrapped Enzymes. Anal. Chem..

[19] Eddington D.T., Beebe D.J. (2004). Flow Control with Hydrogels. Adv. Drug Deliv. Rev..

[20] Beck A., Obst F., Busek M., Grünzner S., Mehner P.J., Paschew G., Appelhans D., Voit B., Richter A. (2020). Hydrogel Patterns in Microfluidic Devices by Do-It-Yourself UV-Photolithography Suitable for Very Large-Scale Integration. Micromachines.

[21] Haefner S., Koerbitz R., Frank P., Elstner M., Richter A. (2018). High Integration of Microfluidic Circuits Based on Hydrogel Valves for MEMS Control. Adv. Mater. Technol..

[22] Haefner S., Rohn M., Frank P., Paschew G., Elstner M., Richter A. (2016). Improved Pnipaam-Hydrogel Photopatterning by Process Optimisation with Respect to Uv Light Sources and Oxygen Content. Gels.

[23] Valentin T.M., DuBois E.M., Machnicki C.E., Bhaskar D., Cui F.R., Wong I.Y. (2019). 3D Printed Self-Adhesive PEGDA–PAA Hydrogels as Modular Components for Soft Actuators and Microfluidics. Polym. Chem..

[24] Champeau M., Heinze D.A., Viana T.N., de Souza E.R., Chinellato A.C., Titotto S. (2020). 4D Printing of Hydrogels: A Review. Adv. Funct. Mater..

[25] Distler T., Boccaccini A.R. (2020). 3D Printing of Electrically Conductive Hydrogels for Tissue Engineering and Biosensors—A Review. Acta Biomater..

[26] Weigel N., Männel M.J., Thiele J. (2021). Flexible Materials for High-Resolution 3D Printing of Microfluidic Devices with Integrated Droplet Size Regulation. ACS Appl. Mater. Interfaces.

[27] Kirchmajer D.M.M., Gorkin R., In Het Panhuis M., Gorkin III R., In Het Panhuis M. (2015). An Overview of the Suitability of Hydrogel-Forming Polymers for Extrusion-Based 3D-Printing. J. Mater. Chem. B.

[28] Frank P., Haefner S., Elstner M., Richter A. (2016). Fully-Programmable, Low-Cost, “Do-It-Yourself” Pressure Source for General Purpose Use in the Microfluidic Laboratory. Inventions.

[29] Cho Y.K., Lee J.G., Park J.M., Lee B.S., Lee Y., Ko C. (2007). One-Step Pathogen Specific DNA Extraction from Whole Blood on a Centrifugal Microfluidic Device. Lab Chip.

[30] Hadwen B., Broder G.R., Morganti D., Jacobs A., Brown C., Hector J.R., Kubota Y., Morgan H. (2012). Programmable Large Area Digital Microfluidic Array with Integrated Droplet Sensing for Bioassays. Lab Chip.

[31] Viktorov V., Mahmud M.R., Visconte C. (2016). Design and Characterization of a New H-C Passive Micromixer up to Reynolds Number 100. Chem. Eng. Res. Des..

[32] Beck A., Obst F., Gruner D., Voigt A., Mehner P.J., Gruenzner S., Koerbitz R., Shahadha M.H., Kutscher A., Paschew G. (2022). Fundamentals of Hydrogel-Based Valves and Chemofluidic Transistors for Lab-on-a-Chip Technology: A Tutorial Review. Adv. Mater. Technol..

[33] Richter A., Howitz S., Kuckling D., Arndt K.F. (2004). Influence of Volume Phase Transition Phenomena on the Behavior of Hydrogel-Based Valves. Sens. Actuators B Chem..

[34] Richter A., Howitz S., Kuckling D., Kretschmer K., Arndt K.-F.F. (2004). Automatically and Electronically Controllable Hydrogel Based Valves and Microvalves - Design and Operating Performance. Macromol. Symp..

[35] Matsuo E.S., Tanaka T. (1988). Kinetics of Discontinuous Volume-Phase Transition of Gels. J. Chem. Phys..

[36] Kuckling D. (2018). Stimuli-Responsive Gels.

[37] Naegel A., Heisig M., Wittum G. (2011). Computational Modeling of the Skin Barrier. Methods Mol. Biol..

[38] Voigt A., Greiner R., Allerdißen M., Richter A., Henker S., Völp M. (2014). Towards computation with microchemomechanical systems. Int. J. Found. Comput. Sci..

[39] Richter A., Klatt S., Paschew G., Klenke C. (2009). Micropumps Operated by Swelling and Shrinking of Temperature-Sensitive Hydrogels. Lab Chip.

[40] Richter A., Klenke C., Arndt K.F. (2004). Adjustable Low Dynamic Pumps Based on Hydrogels. Macromol. Symp..

[41] Haefner S., Frank P., Elstner M., Nowak J., Odenbach S., Richter A. (2016). Smart Hydrogels as Storage Elements with Dispensing Functionality in Discontinuous Microfluidic Systems. Lab Chip.

[42] Haefner S., Frank P., Langer E., Gruner D., Schmidt U., Elstner M., Gerlach G., Richter A. (2017). Chemically Controlled Micro-Pores and Nano-Filters for Separation Tasks in 2D and 3D Microfluidic Systems. RSC Adv..

[43] Ehrenhofer A., Bingel G., Paschew G., Tietze M., Schröder R., Richter A., Wallmersperger T. (2016). Permeation Control in Hydrogel-Layered Patterned PET Membranes with Defined Switchable Pore Geometry - Experiments and Numerical Simulation. Sens. Actuators B Chem..

[44] Richter A., Türke A., Pich A. (2007). Controlled Double-Sensitivity of Microgels Applied to Electronically Adjustable Chemostats. Adv. Mater..

[45] Richter A., Wenzel J., Kretschmer K. (2007). Mechanically Adjustable Chemostats Based on Stimuli-Responsive Polymers. Sens. Actuators B Chem..

[46] Hart R.A., Da Silva A.K. (2013). Self-Optimizing, Thermally Adaptive Microfluidic Flow Structures. Microfluid. Nanofluid..

[47] Mao Z., Yoshida K., Kim J. (2019). wan A Micro Vertically-Allocated SU-8 Check Valve and Its Characteristics. Microsyst. Technol..

[48] Mao Z., Yoshida K., Kim J. (2018). wan Study on the Fabrication of a SU-8 Cantilever Vertically-Allocated in a Closed Fluidic Microchannel. Microsyst. Technol..

[49] Dong L., Agarwal A.K., Beebe D.J., Jiang H. (2007). Variable-Focus Liquid Microlenses and Microlens Arrays Actuated by Thermoresponsive Hydrogels. Adv. Mater..

[50] Richter A., Paschew G. (2009). Optoelectrothermic Control of Highly Integrated Polymer-Based MEMS Applied in an Artificial Skin. Adv. Mater..

[51] Liu X., Liu J., Lin S., Zhao X. (2020). Hydrogel Machines. Mater. Today.

[52] Erol O., Pantula A., Liu W., Gracias D.H. (2019). Transformer Hydrogels: A Review. Adv. Mater. Technol..

[53] Kutscher A., Richter A. Magnetically Hybridized Inkjet-Printed Und Photo-Polymerized Multi-Hydrogel Thermo-Responsive Soft Gripper. Proceedings of the ACTUATOR 2022; International Conference and Exhibition on New Actuator Systems and Applications.

