# Supplementary material for: Fabrication of Chemofluidic Integrated Circuits by Multi-Material Printing
Source: Micromachines (Basel). 2023 Mar 22;14(3):699. doi: 10.3390/mi14030699 (PMC10052728; doi:10.3390/mi14030699)
Supplement: Supplementary file 1 [file micromachines-14-00699-s001.zip › Supplementary Materials Files Description.pdf]

Supplementary Information

Fabrication of chemofluidic integrated circuits by multi-material printing

Alexander Kutscher, Paula Kalenczuk, Mohammed Shahadha, Stefan Grünzner, Franziska Obst, Denise Gruner, Georgi Paschew, Anthony Beck, Steffen Howitz and Andreas Richter

Video S1: Printing with PEG into a hole with diameter 500 µm located in 175 µm thick PMMA foil.

Video S2: Printing with prepolymer solution (450 drops with 550 pl each) on PMMA substrate with 600 µm diameter engraving.

Video S3: Unrestricted fluid flow through engraving in spite of successful hydrogel actuator operation (575 µm diameter valve seat), caused by layer misplacement.

Video S4: Free swelling of hydrogel (400 drops, 60 s exposure time) in water.

Video S5: Recorded video of the fluidic IC-program of the application chip.

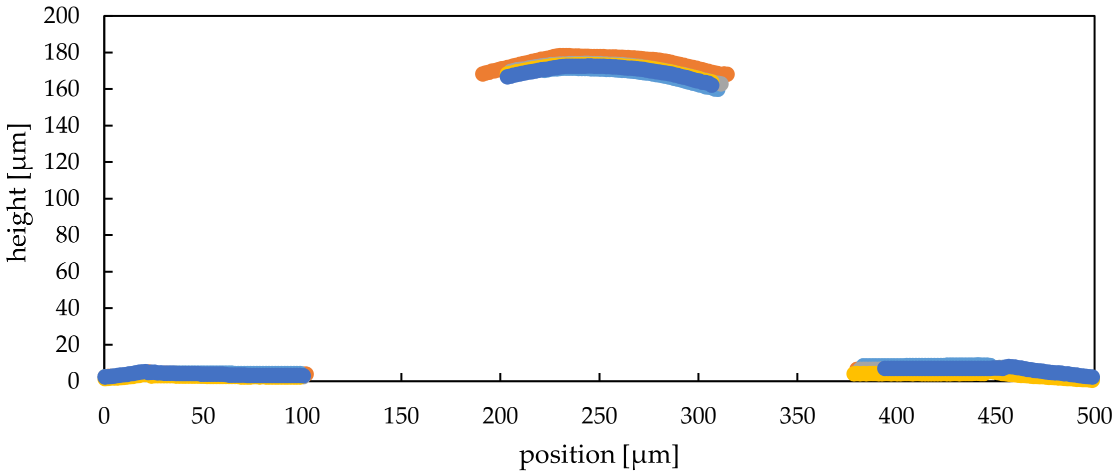

Figure S1: Line profiles of five hydrogels obtained with a confocal scanning microscope µ-Surf. Invalid data due to the slope near the edge was removed.

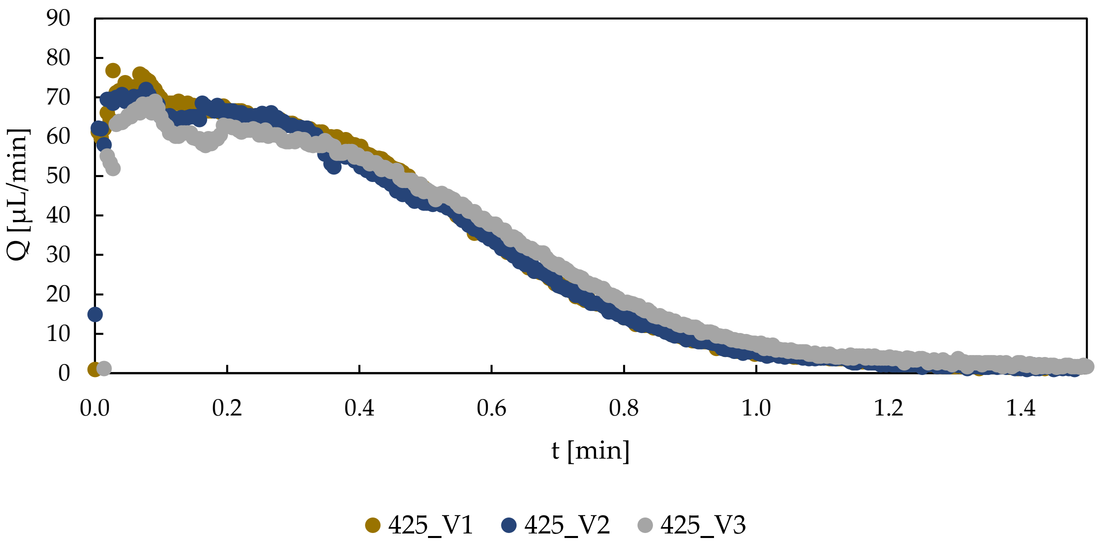

Figure S2: Closing curve diagram for three hydrogels (400 drops, 60s exposure time) in valve seats of 425 µm diameter.
